# Single-cell analysis reveals that Jinwu Gutong capsule attenuates the inflammatory activity of synovial cells in osteoarthritis by inhibiting AKR1C3

**DOI:** 10.3389/fphys.2022.1031996

**Published:** 2022-11-23

**Authors:** Junfeng Guo, Chuyue Tang, Zhao Shu, Junfeng Guo, Hong Tang, Pan Huang, Xiao Ye, Taotao Liang, Kanglai Tang

**Affiliations:** ^1^ State Key Laboratory of Trauma, Burn and Combined Injury, Department of Orthopedics/Sports Medicine Center, Southwest Hospital, Third Military Medical University, Chongqing, China; ^2^ The First Affiliated Hospital of Chongqing Medical University, Chongqing, China; ^3^ Department of Stomatology, The 970th Hospital of the Joint Logistics Support Force, Yantai, China

**Keywords:** Jinwu Gutong capsule, synovitis, osteoarthritis, scRNA-seq, AKR1C3

## Abstract

Jinwu Gutong capsule (JGC) is a traditional Chinese medicine formula for the treatment of osteoarthritis (OA). Synovitis is a typical pathological change in OA and promotes disease progression. Elucidating the therapeutic mechanism of JGC is crucial for the precise treatment of OA synovitis. In this study, we demonstrate that JGC effectively inhibits hyperproliferation, attenuates inflammation, and promotes apoptosis of synovial cells. Through scRNA-seq data analysis of OA synovitis, we dissected two distinct cell fates that influence disease progression (one fate led to recovery while the other fate resulted in deterioration), which illustrates the principles of fate determination. By intersecting JGC targets with synovitis hub genes and then mimicking picomolar affinity interactions between bioactive compounds and binding pockets, we found that the quercetin-AKR1C3 pair exhibited the best affinity, indicating that this pair constitutes the most promising molecular mechanism. *In vitro* experiments confirmed that the expression of AKR1C3 in synovial cells was reduced after JGC addition. Further overexpression of AKR1C3 significantly attenuated the therapeutic efficacy of JGC. Thus, we revealed that JGC effectively treats OA synovitis by inhibiting AKR1C3 expression.

## Introduction

Osteoarthritis (OA) is the most common age-related chronic degenerative whole-joint disease and affects more than 300 million people worldwide (Choi et al., 2019; Boer et al., 2021). OA imposes a severe social and economic burden, and its total costs are estimated to equal 1%–2.5% of a country’s gross domestic product (GDP) (Hiligsmann et al., 2013; Brown et al., 2021). The main pathological features of OA are cartilage degeneration and synovial inflammation (Sellam and Berenbaum, 2010). Increasing evidence indicates that synovial inflammation not only is directly linked to clinical symptoms such as joint swelling and inflammatory pain but also increases cartilage injury (Atukorala et al., 2016; Labinsky et al., 2020). Thus, inhibiting synovitis is a crucial aspect of preventing OA development.

The current treatments for synovitis mainly include nonsteroidal anti-inflammatory drugs (NSAIDs) and glucocorticoids (GSs), but their effects are often short-lived and may even lead to a greater degree of cartilage loss (Conaghan et al., 2019; Pontes-Quero et al., 2021). Jinwu Gutong capsule (JGC) is a traditional botanical formula widely used in China for OA treatment and is widely believed to have considerable potential with respect to clinical efficacy (Zhao et al., 2022). Indeed, the combined application of JGC with NSAIDs or GS can significantly improve the efficacy of OA treatment. However, the pharmacological mechanism of JGC remains unclear and warrants further research.

Single cell sequencing provides insights into the underlying mechanisms of OA development. Early research mainly focused on cartilage degeneration: Tang et al. identified seven molecularly defined populations of chondrocytes in the human OA cartilage (Ji et al., 2019); Jeon et al. (2017) found that p16^INK4a^ positive senescent chondrocytes contribute to the development of spontaneous and injury-induced OA. In recent years, people have increasingly recognized the important role of synovitis in the development of OA. Nanus et al. (2021) illustrated that there are distinct synovial fibroblast subsets in early OA and end-stage OA. Knights et al. (2022) displayed Prg4^hi^ lining fibroblasts secrete Rspo2, which drives pathological joint crosstalk after injury.

In this study, we demonstrate the therapeutic effect of JGC on synovial inflammation and hyperplasia. A single-cell synovial atlas was produced, which allowed an in-depth exploration of the synovial microenvironment. Further transcriptional dynamics analysis revealed a cell fate decision mechanism that affects disease progression and recovery. We also identified the target of JGC in treating OA synovitis and verified this target through computer simulations and biological experiments.

## Materials and methods

### Preprocessing of Jinwu Gutong capsule

Commercial JGC (specification: 0.5 g per pill) was purchased from Guizhou SSLF Pharmaceutical Co., Ltd. (Guizhou, China, approval number: Z20043621). According to the literature (Sridhar et al., 2021), JGC was powdered and extracted using a Soxhlet extractor with 6 times the amount of 90% ethanol. The solvent was then concentrated using an electrically heated blast drying oven at 45°C. Subsequently, the concentrate was lyophilized with a freeze dryer and weighed. The JGC extract was dissolved in DMSO (20 mg/ml) and stored at −80°C for later use.

### Cell culture

The human synovial cell line SW982 was kindly provided by Procell Life Science and Technology Co., Ltd. (Wuhan, China). SW982 cells have been shown to possess characteristic features similar to synovial fibroblasts which makes them an ideal tool to study synovitis in OA (Karuppagounder et al., 2022). The cells were cultured in DMEM/Ham’s F12 medium (DMEM/F12; HyClone, Logan, UT, United States) with 10% fetal bovine serum (PAN Biotech, Aidenbach, Germany) and 1% penicillin/streptomycin (Gibco, Grand Island, NY, United States).

### Detection of cell proliferation

The cell proliferative capacity was determined by Cell Counting Kit-8 assays (CCK-8, Biosharp, Guangzhou, China). Cells (10,000/well) were plated in 96-well plates, and DMSO, CTGF or JGC was added according to the experimental design. CTGF is a pro-inflammatory cytokine, that is, upregulated in OA and contributes to synovial hyperplasia (MacDonald et al., 2021). The working concentration of CTGF was 25 ng/ml, and that of JGC was 20 μg/ml. After 24 h, the supernatant was replaced with CCK-8 working solution, and the absorbance at 450 nm was measured.

### Apoptosis detection

An Annexin V-FITC Assay Kit (Merck, NJ, United States) was used to detect apoptosis in synovial cells. The cells were plated in 6-well plates (50,000/well) and processed as described above. After 24 h, the cells were dissociated and stained according to the instructions provided with the kit. In brief, cells were digested with trypsin, washed gently with PBS, resuspended in buffer solution to 1 × 10^6^ cells/ml. Then 5 μl Annexin V-FITC was added, and the mixture was incubated in the dark for 5 min 5 μl propidium iodide (PI) was added to the cells before analyzed. We measured the proportion of FITC(+) cells by flow cytometry.

### Data sources and processing

Single-cell sequencing data for synovial cells were downloaded from the GEO database (no. GSE176308), and 10X genomics data were loaded into the R package Seurat (v4.0.2). Synovial cells were obtained from 4 patients with early-stage OA (both painful and non-painful sites) and 4 patients with end-stage OA (painful sites) (Nanus et al., 2021). Cell quality control was applied to remove low-quality cells with less than 300 detected genes or with more than 10% mitochondrial genes. After normalizing the data, the cells were dimensionally reduced and clustered according to the top 2,000 highly variable genes. The FindIntegrationAnchors algorithm found a set of anchors between Seurat objects from different patients. These anchors could be used to integrate the objects using the IntegrateData function. Harmony package (v1.0) was used to remove the batch effect, the diversity clustering penalty parameter was set to 2 and the ridge regression penalty parameter was set to 1.

### Pseudotime analysis

The dynamic states of synovial cells were assessed using the Monocle algorithm (v2.18.0). Monocle uses an unsupervised algorithm to order whole-transcriptome profiles of single cells and produce a ‘trajectory’ of an individual cell’s progress through differentiation. We applied the “reduceDimension” function to compute the CellDataSet object as a lower dimensional trajectory. The Discriminative Dimensionality Reduction with Trees (DDRTree) method was chosen for its ability to reduce dimensionality while discriminating between different data points. Following dimension reduction, the two features with the most significant amount of information were extracted and used as the coordinate axes to visualize the trajectory. Branched expression analysis modeling (BEAM) was performed to identify genes with branch-dependent expression and thus elucidate fate decision mechanisms.

### Cell cycle analysis

Independent cell cycle analysis was performed for each synovial cell. The “CellCycleScoring” function in the Seurat package was used to assign cell cycle scores according to S- and G2/M-phase genes, which were identified following procedures described in a previous study (Kan et al., 2022). The number of control features selected from the same bin per analyzed feature was set to 100 and the random seed was set to 1. The cells were classified into G1, S, and G2/M phases based on the maximal score of each cell cycle phase program.

### Jinwu Gutong capsule target prediction

We obtained information regarding the main raw materials from the JGC drug manual. Information about the main active ingredients of these raw materials was obtained from the relevant literature (Supplementary Table S2). The SDF format files of molecular structures were downloaded from the Pubchem database (https://pubchem.ncbi.nlm.nih.gov/). Targets of these molecular structures were predicted using the SwissTargetPrediction database (http://www.swisstargetprediction.ch/) (Daina et al., 2019). The species was confined to “*Homo sapiens*”, and the predicted targets with a probability more than 0.3 were included in this study.

### Molecular docking

Macromolecular structures were downloaded from the RCSB PDB database (https://www.rcsb.org), and biological ligands were accessed from PubChem database. PDB files were converted to the PDBQT format. We used AutoDockTools software to search for possible active pockets, removed all water molecules and assigned hydrogen polarities. AutoDock Vina was employed to conduct molecular docking between the active ingredients and targets, then took the conformation with the highest docking score (Affinity). Finally, we used the PyMOL software to visualize the results of molecular docking.

### Statistical analysis

Bilateral tests were performed for all statistical tests. A *p*-value lower than 0.05 was considered to indicate statistical significance. R software version 4.0.2 (https://www.r-project.org/) was used for the analysis. The following R language packages were used in this study: “dplyr”, “Seurat”, “monocle”, “monocle”, and “iTALK”. The “drug-material-target” network was visualized using Cytoscape_3.7.2 (https://cytoscape.org).

## Results

### Jinwu Gutong capsule exerts ideal therapeutic effects on reducing inflammation and hyperplasia of synovial cells

JGC is widely used for OA treatment with ideal clinical efficacy. According to the instructions, the main raw materials of JGC include *Cibotium barometz* (CB [Cyatheaceae; *Cibotium barometz* (L.) J. Sm]), Epimedium (ED [Berberidaceae; *Epimedium sagittatum* (Siebold & Zucc.) Maxim]), Clematidis radix (CR [Ranunculaceae; Clematis chinensis Osbeck]), Zaocys dhumnades (ZD [Colubridae]), *Achyranthes bidentata* Blume (ABB [Amaranthaceae; *Achyranthes bidentata* Blume]), Chaenomeles sinensis (CS [Rosaceae; *Pseudocydonia sinensis* (Dum.Cours.) C.K. Schneid]), *Pueraria lobata* (PL [Fabaceae; *Pueraria montana* var. lobata (Willd.) Maesen & S.M. Almeida ex Sanjappa & Predeep]), *Curcuma longa* (CL [Zingiberaceae; *Curcuma longa* L., Sp. Pl.: 2 (1753)]), *Psoralea corylifolia* Linn. (PCL [Fabaceae; *Cullen corylifolium* (L.) Medik]), and *Campanumoea javanica* bl (CJB [Campanulaceae; *Codonopsis javanica* (Blume) Hook. f. & Thomson, Ill. Himal. Pl. t.16 B (1855)]). Certain materials (ED, ABB, CS, PL, CL, and CR) reportedly have significant anti-inflammatory and antioxidant activities, and the aqueous extract of CR exerts a good anti-osteoarthritis effect (Cheng et al., 2013; Lin et al., 2019; Cheng et al., 2020; Jeon et al., 2020; Lin et al., 2021; Razavi et al., 2021). The reasonable compatibility of these materials guarantees curative efficacy.

**FIGURE 1:**
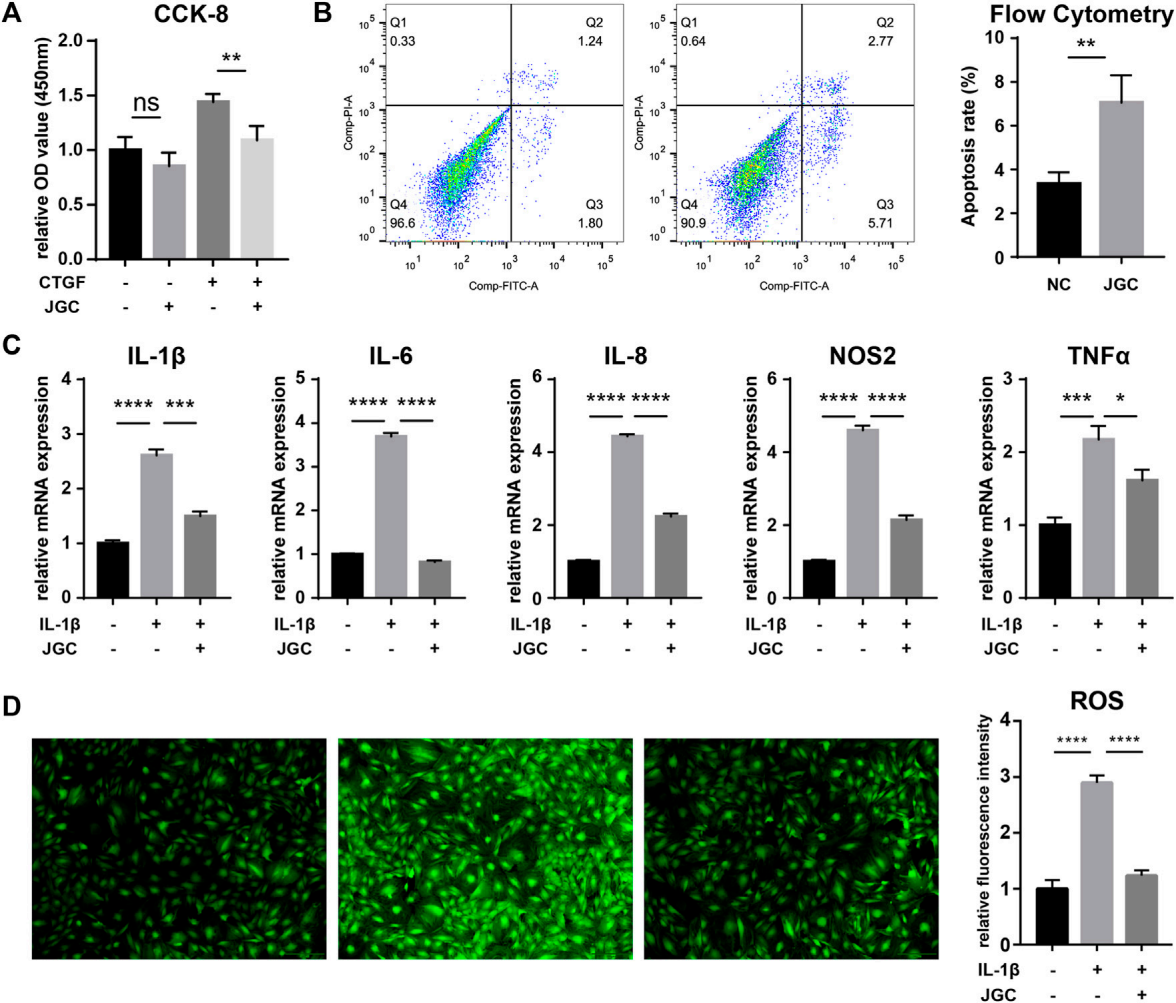
Therapeutic effect of JGC on synovitis. **(A)** CCK-8 assay showing the effect of JGC on cell proliferation. **(B)** Flow cytometry showing the effect of JGC on apoptosis. **(C)** PCR showing that JGC effectively inhibits synovial inflammation. **(D)** JGC clearly reduces the intracellular ROS levels.

Synovial tissue shows discordant hyperplasia and inflammation during OA progression. The human synovial cell line SW982 was treated with JGC to assess the effect of this drug on synovial hyperplasia. In normal synovial cells, the inhibition of proliferation by JGC was not significant, indicating tolerable drug toxicity. We then induced hyperproliferation using the growth factor CTGF, and JGC exerted a more pronounced inhibitory effect on the proliferation of active synovial cells (Figure 1A). Flow cytometry showed that the proportion of FITC(+) synovial cells was significantly increased, showing the apoptosis-promoting effect of JGC on SW982 cells (Figure 1B). The inflammatory cytokine IL-1β was applied to induce intense inflammation in synovial cells. Although the expression levels of numerous inflammatory genes (IL-1β, IL-6, IL-8, NOS2, and TNF-α) were clearly increased, JGC treatment significantly reversed the increase in expression caused by inflammatory stimulation (Figure 1C). We also found similar trends for the intracellular reactive oxygen species (ROS) levels: inflammation led to increased ROS levels in SW982 cells, and this increase was relieved after JGC addition (Figure 1D). These results confirm the therapeutic effect of JGC on synovitis *in vitro*.

### Cellular composition and communication of synovial microenvironment in osteoarthritis

**FIGURE 2:**
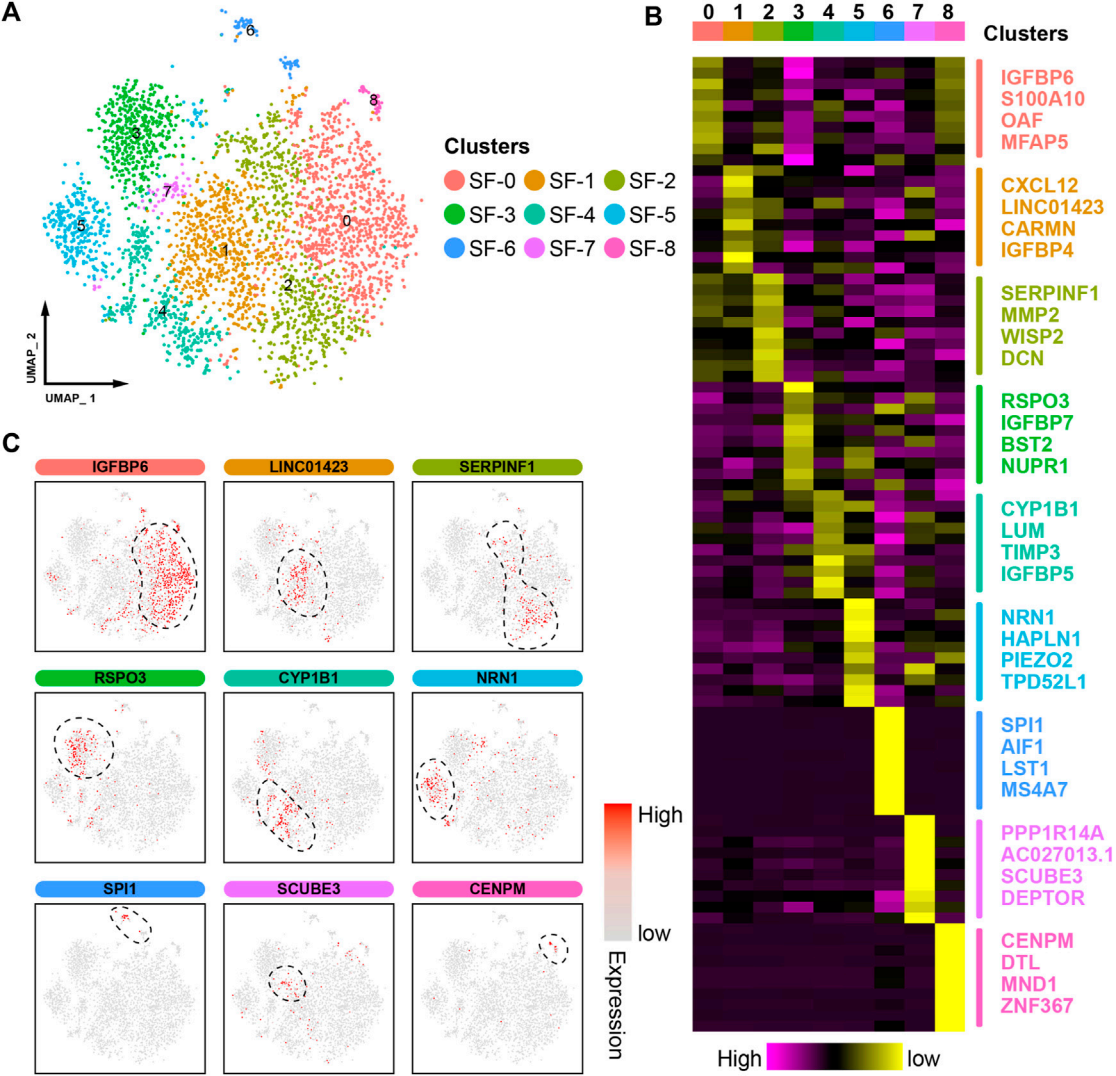
ScRNA-seq profiling of synovitis microenvironments. **(A)** A uniform manifold approximation and projection (UMAP) plot showing the color-coded cell clusters in the synovitis microenvironment. **(B)** Heatmap showing the marker gene expression in the different cell clusters. **(C)** UMAP plot showing the marker gene expression in the different cell clusters.

To deeply dissect the molecular mechanism of JGC in the treatment of OA synovitis, scRNA-seq data from 4145 synovial fibroblasts (SFs) were examined in this study. SFs were clustered into nine color-labeled subsets based on their unbiased transcriptome signatures (Figure 2A). The cell cluster properties were preliminarily assessed based on cluster-specific markers (Figures 2B,C; Supplementary Figure S1; Supplementary Table S1): the cells in SF-0 expressed high levels of IGFBP6, MFAP5, and SEMA3C, indicating their high proliferative capacity; the cells in SF-1 overexpressed CXCL12 and ID1, suggesting a stronger inflammatory stimulus; the cells in SF-2 expressed MMP2 and WISP2, which play decisive roles in fibrosis; the cells in SF-5 showed relatively high expression of Piezo2, a mechanosensitive channel; the cells in SF-6 expressed RNASE1, indicating decreased adhesion to cartilage; the cells in SF-7 expressed genes critical for synovial angiogenesis (expressing SCUBE3); and the cells in SF-8 expressed relatively high levels of a cell cycle-related gene (CENPM).

**FIGURE 3:**
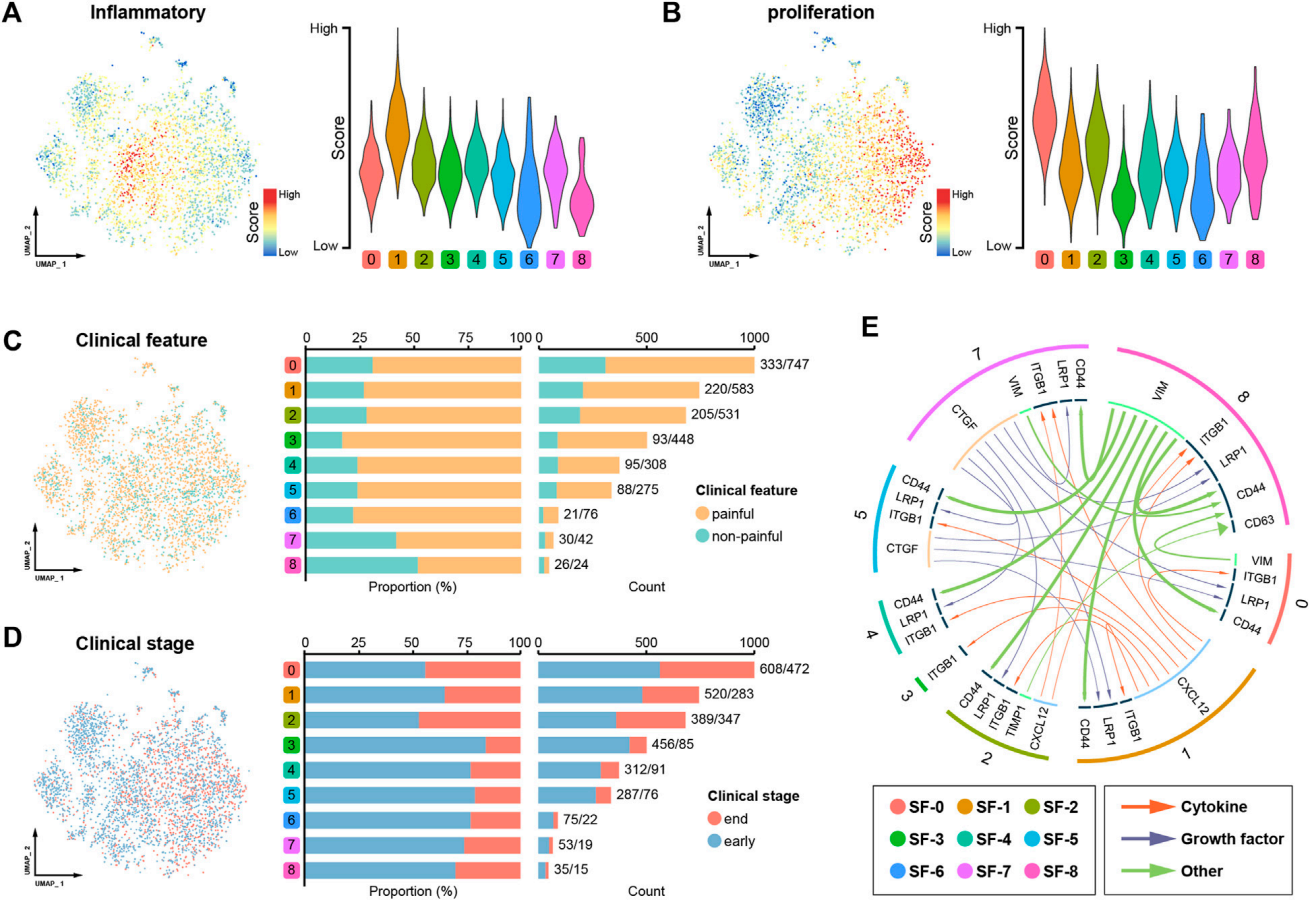
Assessment of the synovial microenvironment and intercellular communication. **(A)** UMAP plot showing the level of inflammation in the different cell clusters. **(B)** UMAP plot showing the proliferation ability of the different cell clusters. **(C)** Distribution of cells from patients with or without pain. **(D)** Distribution of cells from early- and end-stage OA patients. **(E)** Cell‒cell communication in the synovial microenvironment.

We further calculated module scores to assess their inflammatory and proliferative activities, which are the two most prominent pathological features of synovitis. Consistent with the abovementioned results, the SF-1 synovial cells showed the highest level of inflammation, whereas the SF-0 cells exhibited an excessive proliferative capacity (Figures 3A,B). Overall, the proportions of cells from patients with or without pain, according to clinical information, did not significantly differ among the clusters; however, higher proportions of cells in SF-0, SF-1, and SF-2 were obtained from end-stage OA patients (Figures 3C,D). A cell‒cell communication analysis revealed complex ligand‒receptor interactions in the synovial microenvironment, and intercellular crosstalk was mainly divided into cytokines, growth factors and others (Figure 3E). Based on the cytokine categories, the synovial cells in SF-1 expressed higher levels of CXCL12, which interacts with the ITGB1 receptor of surrounding cells to regulate proinflammatory cytokine production (Kong et al., 2020). The growth factor category revealed that CTGF secreted by SF-7 cells interacts with LRP1, which is highly expressed on the surface of cells in other clusters, to induce pathological progression (Schnieder et al., 2020).

### Transcriptional dynamics analysis reveals the regulation of synovial cell fate decisions

**FIGURE 4:**
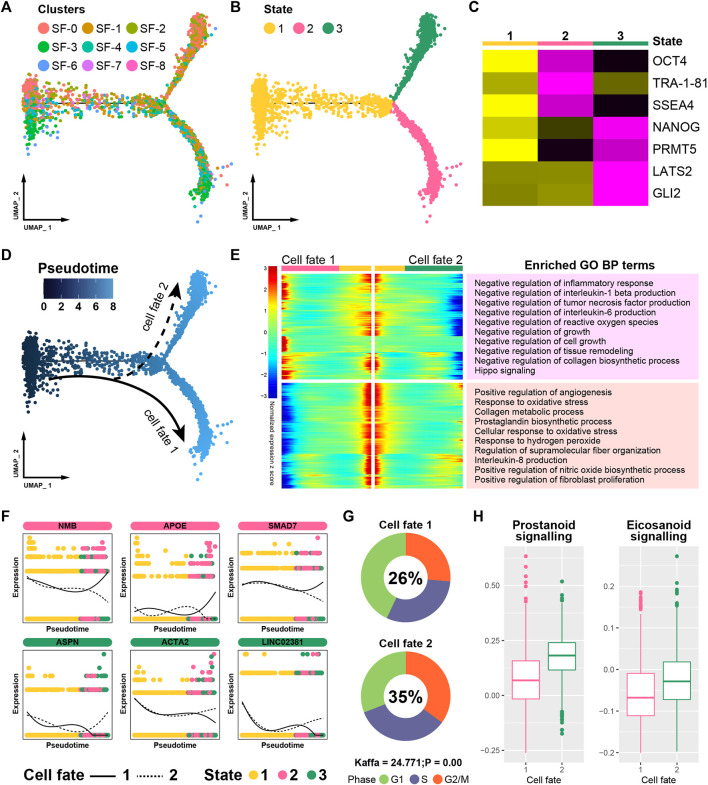
Pseudotime analysis of the synovium. **(A)** Trajectory plot of distinct cell clusters. **(B)** Trajectory plot of pseudotime states. **(C)** Trajectory heatmap of different cell states. **(D)** Trajectory plot of different cell fates. **(E)** Trajectory heatmap of different cell fates. **(F)** Branch trend curves of crucial genes. **(G)** Cell cycle distribution of different fates. **(H)** The activation levels of “Eicosanoid Signaling” and “Prostanoid Signaling”.

The Monocle pseudotime algorithm was used to profile the fate trajectory of synovial cells. The cells were dimensionally descended and arranged in a typical dendritic shape (Figure 4A), and the fate trajectory was divided into three cell states based on bifurcation points (Figure 4B, state 1 to state 3). By comparing the gene patterns in distinct cell states, we found certain classical progenitor/stem cell markers to be significantly overexpressed in cell state 1 (OCT-4, TRA-1-81, SSEA4, NANOG, etc.). Thus, cell state 1 was defined as the origin of the trajectory (Figure 4C), and the synovial cells gradually differentiated into two distinctive fates as the trajectory progressed (Figure 4D).

We screened for “branch-dependent” genes that changed as the cell fate developed and divided these genes into two gene modules. A Gene Ontology (GO) enrichment analysis of “branch-dependent” genes helped annotate the cellular properties across different cell fates (Figure 4E). Certain functions that are beneficial to synovitis recovery were significantly activated in cell fate 1 (e.g., negative regulation of the inflammatory response and cell growth). However, some terms that suggest pathogenesis were enhanced in cell fate 2 (such as positive regulation of angiogenesis). The expression patterns of some canonical synovitis regulators were further assessed, and certain restorative genes (such as NMB, APOE and SMAD7) were highly expressed in cell fate 1 but decreased in cell fate 2. In addition, some pathogenic genes, such as ASPN and ACTA2, showed completely contrary trends (Figure 4F). A cell cycle analysis showed that the proportion of actively proliferating cells (G2/M) was significantly higher in cell fate 2, indicating likely tissue hyperplasia (Figure 4G). What’s more, the two pathways associated with pain (prostanoid and eicosanoid signaling) showed increased activation in cell fate 2, suggesting that these cells were more likely to induce clinical symptoms (Figure 4H). In summary, these results suggest that cells in cell fate 1 contribute to recovery and that cells in cell fate 2 lead to synovitis progression.

### Jinwu Gutong capsule treats synovitis by inhibiting AKR1C3

**FIGURE 5:**
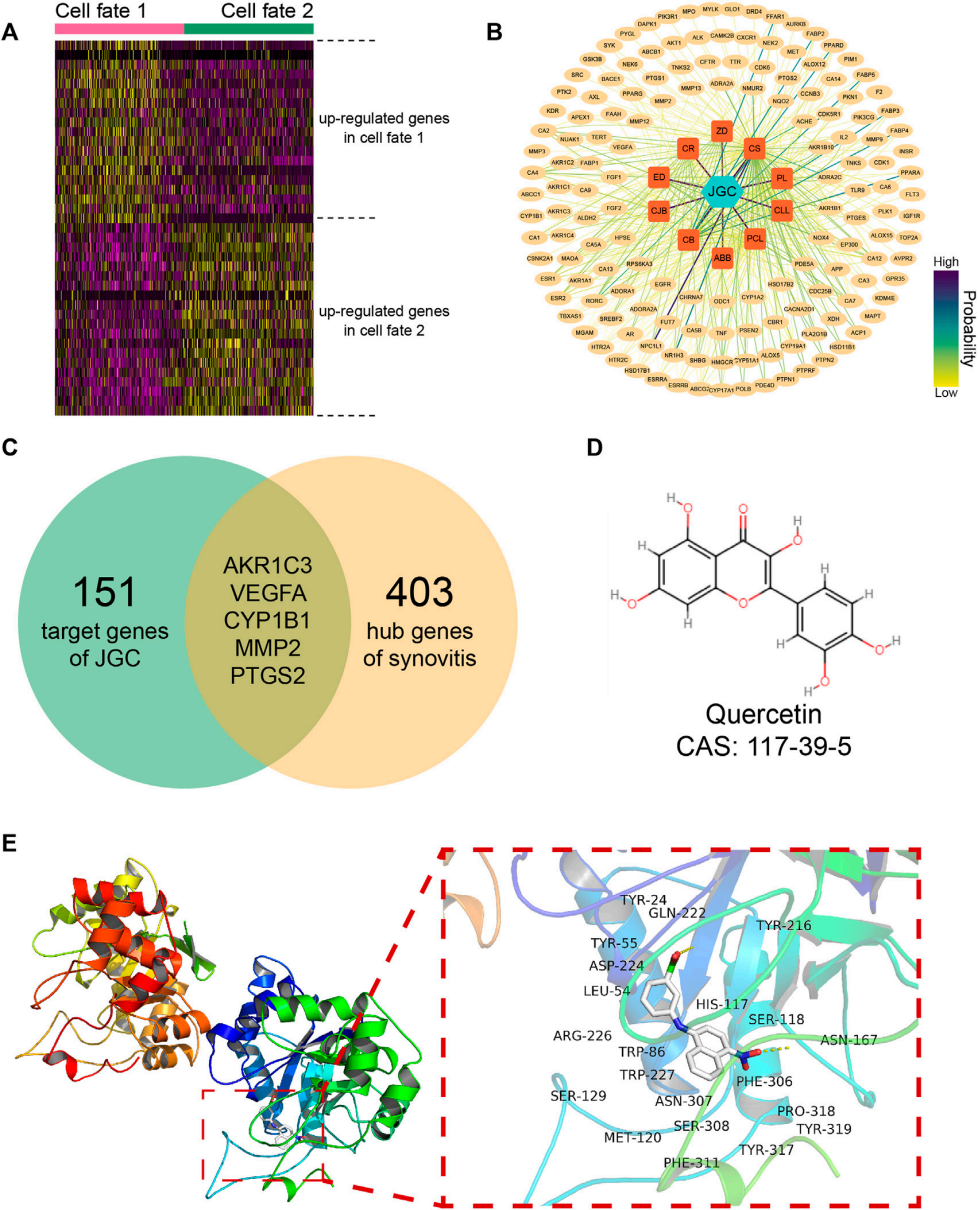
JGC treats synovitis by inhibiting AKR1C3. **(A)** Heatmap showing differentially expressed genes among distinct cell fates. **(B)** Network showing predicted targets of JGC. **(C)** Venn diagram showing the intersection of JGC targets with hub genes of synovitis. **(D)** Molecular structure of quercetin. **(E)** Molecular docking pattern of the quercetin-AKR1C3 pair.

A differential expression analysis between the two cell fates identified a total of 403 key synovitis genes, including 195 and 208 upregulated genes in cell fate 1 and cell fate 2, respectively (Figure 5A). Furthermore, by summarizing previous research results, we collected 122 bioactive molecules from the raw materials of JGC (Supplementary Table S2). Subsequently, 151 potential targeting relationship pairs were predicted from the SwissTargetPrediction database (Supplementary Table S3), and a “drug-material-target” network was generated to visualize the potential therapeutic mechanism (Figure 5B). By taking the intersection of JGC targets with key genes of synovitis, five promising functional targets (AKR1C3, VEGFA, CYP1B1, MMP2, and PTGS2) were obtained (Figure 5C). Molecular docking was performed to simulate the interaction between bioactive compounds and binding pockets, which revealed a molecular basis for this picomolar affinity (Supplementary Figure S2). The quercetin-AKR1C3 pair exhibited the best affinity, indicating that this pair constitutes the most promising molecular mechanism (Figures 5D,E; Table 1).

**TABLE 1:** Molecular docking results.

Bioactive compounds	Targets	affinity (kcal/mol)
quercetin	AKR1C3	−10.1
syringetin	CYP1B1	−9.3
apigenin	CYP1B1	−8.3
quercetin	MMP2	−8.2
quercetin	CYP1B1	−7.9
chlorogenic acid	MMP2	−7.7
apigenin	PTGS2	−7.7
icariside F2	VEGFA	−2.3

**FIGURE 6:**
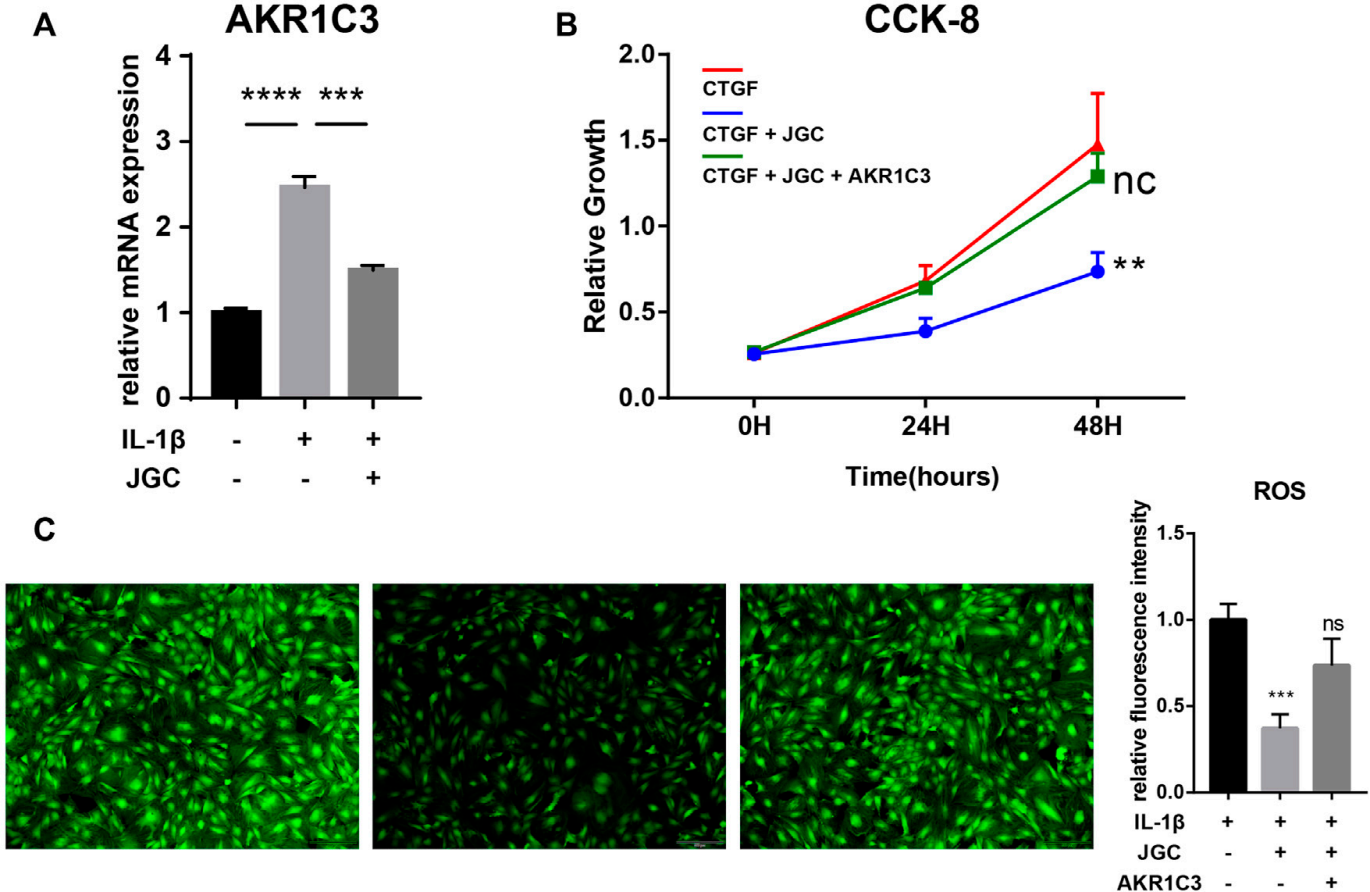
Rescue experiments of AKR1C3. **(A)** PCR showing that AKR1C3 is inhibited by JGC. **(B)** CCK-8 assay showing that AKR1C3 overexpression attenuates the JGC-mediated inhibition of cell proliferation. **(C)** ROS staining showing that AKR1C3 overexpression counteracts the anti-inflammatory effect of JGC.

Further PCR results confirmed the hypothesis that AKR1C3 expression was elevated in inflamed synovial cells and effectively inhibited by the addition of JGC (Figure 6A). Rescue experiments were performed to characterize the regulatory relationship. AKR1C3 overexpression significantly attenuated the JGC-induced inhibitory effect on synovial cell proliferation (Figure 6B). Similarly, the anti-inflammatory effect of JGC on synovial cells was clearly counteracted by AKR1C3 overexpression (Figure 6C). Taken together, our findings suggest that JGC treats synovitis in osteoarthritis by inhibiting AKR1C3.

## Discussion

OA is a chronic degenerative disease that involves pain and disability, resulting in poor quality of life (Xie et al., 2021). Severe synovitis is one of the typical pathological features of OA and leads to disease progression (Jin et al., 2011; Zhang et al., 2022). Certain botanical drugs, such as saponins and kaempferol, have been shown to act as effective therapeutics in inflammatory diseases (Devi et al., 2015; Dong et al., 2019). As a traditional botanical formula, JGC has been widely used in clinical practice and exerts good curative effects on OA synovitis. Thus, elucidating the molecular mechanism of JGC has important academic value and broad application prospects.

The pathological changes occurring in the OA synovium mainly include inflammation, hyperplasia and fibrosis, all of which usually coexist (Kuang et al., 2020). Our study shows that JGC effectively inhibits the expression of proinflammatory factors in synovial cells and reduces the intracellular ROC levels in these cells. Furthermore, JGC restrained the overproliferation of and induced apoptosis in synovial cells. These results confirm the therapeutic effect of JGC on synovitis at the cellular level, which complements the results from previous studies.

A pseudotime analysis revealed the transcriptional dynamics and cell trajectory fates of synovial cells. In addition to the inflammation-, proliferation-, and fibrosis-related terms mentioned above, we found that the Hippo pathway was significantly activated in cell fate 1. The cells in cell fate 1 were identified as synovitis repair cells, and certain previous studies support our conclusion that activation of the Hippo pathway by verteporfin significantly reduces the severity of synovitis (Caire et al., 2021; Symons et al., 2022). Certain key genes (APOE and SMAD7) were found to silence cell fate 2. Apolipoprotein E, a major apoprotein of the chylomicron, inhibits synovial activation and ectopic bone formation (de Munter et al., 2016); in contrast, Smad7 loss promotes synovial inflammation and fibrosis (Blaney Davidson et al., 2006; Zhou et al., 2018). Moreover, the expression of several disease progression genes (ASPN, ACTA2 and LINC02381) was increased in cell fate 2 (Yang et al., 2018; Wang and Zhao, 2020; Wei et al., 2021). Joint pain is the predominant symptom of OA. “Eicosanoid Signaling” and “Prostanoid Signaling” are thought to be the main contributors to OA pain (Sanchez-Lopez et al., 2022). Several enzymes of the eicosanoid receptors are well-recognized targets of anti-inflammatory drugs that can reduce synovial inflammation (Korotkova and Jakobsson, 2014). Interestingly, our study found that cells in fate 2 were more active in both pathways. This finding indicated that as synovial cells progress toward fate 2, the patient’s pain symptoms will likely become more severe. Overall, the consistency of our results with those from previous studies bolsters the reliability of our findings on cell fate determination.

We found that quercetin, an active component of JGC, well matched the active pocket of AKR1C3, and a PCR analysis confirmed a regulatory relationship. The steroidogenic enzyme AKR1C3 plays an important role in many diseases, such as prostaglandin disorder, metastatic breast tumors and atopic dermatitis (Mantel et al., 2012; Evans et al., 2019; Li et al., 2020). AKR1C3 mediates hyperproliferation, oxidative stress and drug resistance in various tissues (González-Muniesa et al., 2013; Yepuru et al., 2013; Thoma, 2015). Although AKR1C3 is a promising therapeutic target, no AKR1C3-targeting drugs have been approved for clinical use to date (Pippione et al., 2017). As a natural product, quercetin has been extensively evaluated for its efficacy and pharmacological safety (Hu et al., 2017; Ulusoy and Sanlier, 2020; Lai and Wong, 2021; Yan et al., 2022). Our study verifies the therapeutic effect of quercetin on OA synovitis by targeting AKR1C3, which further broadens the potential application of quercetin.

This study has some limitations. There were relatively few synovitis scRNA-seq samples and a lack of corresponding chondrocytes and subchondral bone samples. Analysis of additional samples would be conducive to eliminating the heterogeneity caused by individual differences. Simultaneous analysis of data from multiple tissues (synovium, cartilage, subchondral bone) is beneficial to deepen our understanding of OA disease process.

In summary, our study confirms the beneficial influence of JGC in OA synovitis and thus shows that JGC effectively suppresses inflammation and hyperproliferation in synovial cells. An in-depth profiling of the synovitis microenvironment and transcriptional dynamics revealed two distinct cell fates that resolve or advance the disease. We also identified the pharmacological mechanism of the quercetin-AKR1C3 pair of JGC in the treatment of OA synovitis. These efforts will help researchers better elucidate OA synovitis and improve treatment outcomes.

## Data Availability

The raw ordinary scRNA-seq data for synovitis can be accessed from GEO (GSE176308). The software programs and packages used to analyze the dataset are freely available. Further inquiries can be directed to the corresponding authors.

## Abbreviations

OA, osteoarthritis; JGC, Jinwu Gutong capsule; GDP, gross domestic product; NSAIDs, nonsteroidal anti-inflammatory drugs; GSs, glucocorticoids; DMSO, dimethyl sulfoxide; CCK‐8, Cell Counting Kit‐8; BEAM, branched expression analysis modeling; CB, Cibotium barometz; ED, Epimedium; CR, Clematidis radix; ZD, Zaocys dhumnades; ABB, *Achyranthes* bidentata Blume; CS, Chaenomeles sinensis; PL, Pueraria lobata; CL, curcuma longa; PCL, psoralea corylifolia Linn.; CJB, Campanumoea javanica bl; FITC, fluorescein isothiocyanate isomer; ROS, reactive oxygen species; SFs, synovial fibroblasts; PCR, polymerase chain reaction; UMAP, uniform manifold approximation and projection.
