# Supplementary material for: Single-cell analysis reveals that Jinwu Gutong capsule attenuates the inflammatory activity of synovial cells in osteoarthritis by inhibiting AKR1C3
Source: Front Physiol. 2022 Nov 23;13:1031996. doi: 10.3389/fphys.2022.1031996 (PMC9727177; doi:10.3389/fphys.2022.1031996)
Supplement: Supplementary file 1 [file DataSheet1.docx]

Jinwu Gutong Capsule attenuates synovitis in osteoarthritis by inhibiting AKR1C3

**supplementary materials**


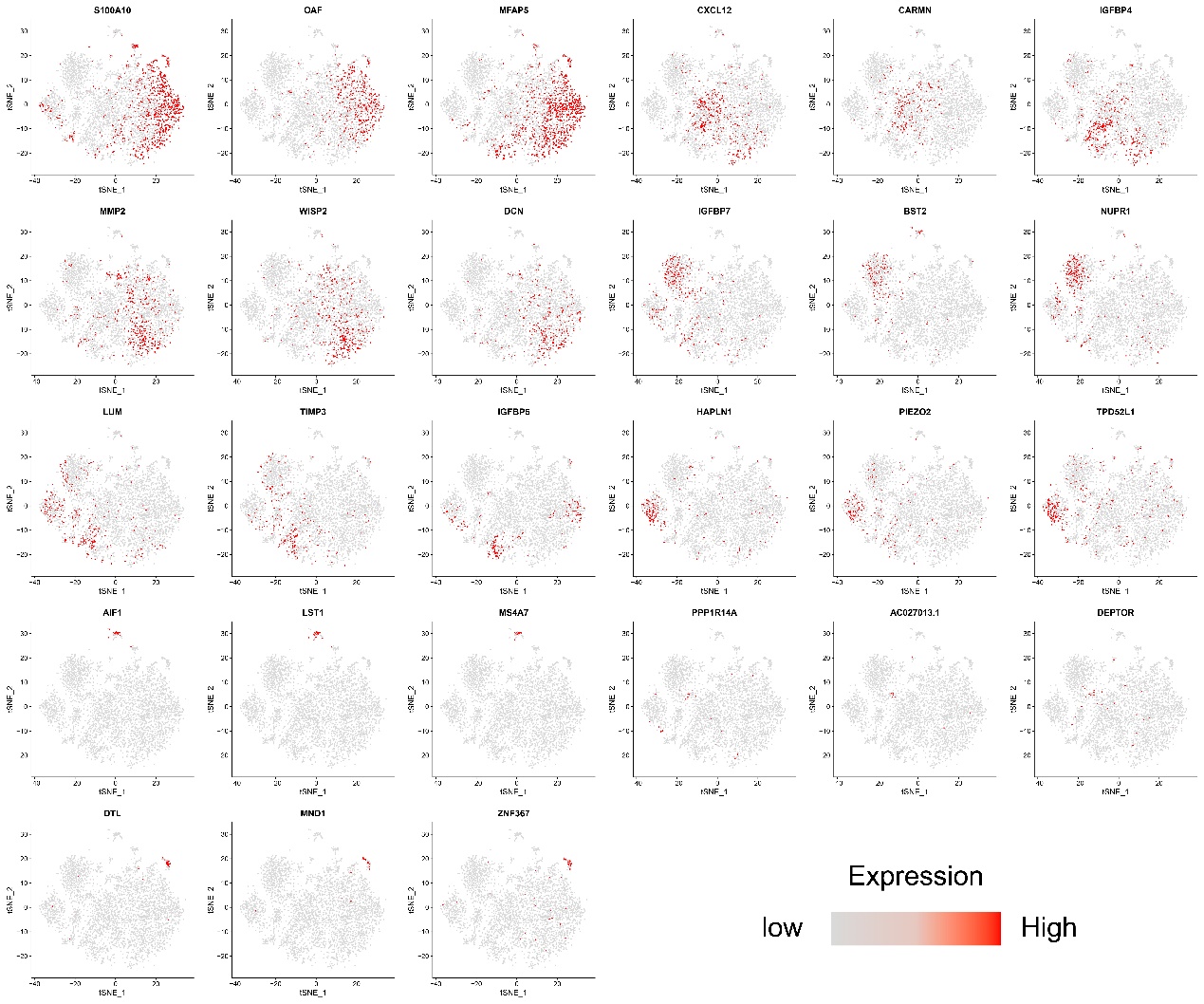


**Figure S1.** UMAP plots showing cluster-specific markers.


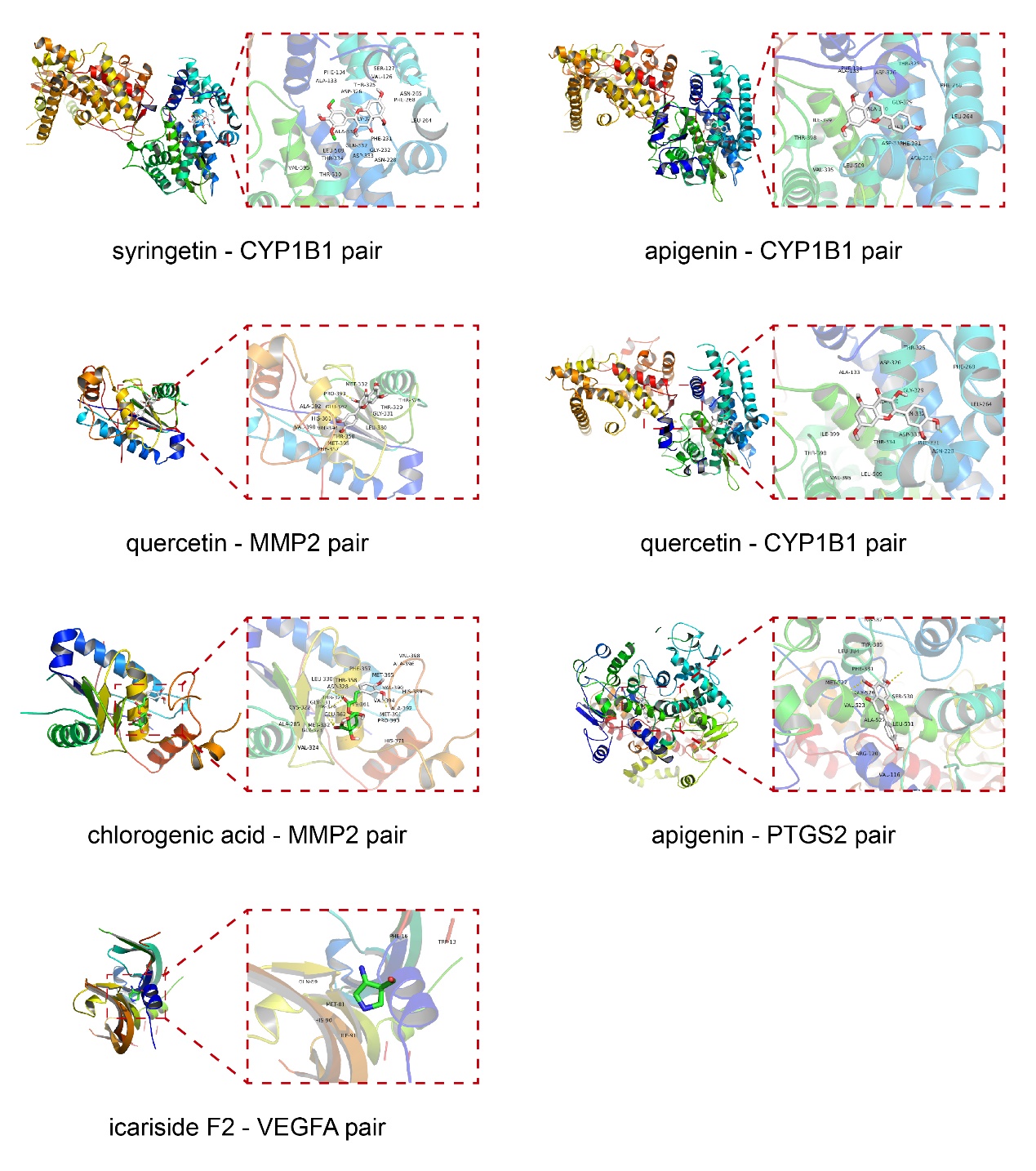


**Figure S2.** Molecular docking results.

**Table S1.** The specific markers for the 9 cell clusters in synovial tissues.

| cluster identity | marker genes | references |
| --- | --- | --- |
| 0 | IGFBP6, MFAP5, SEMA3C | [1-3] |
| 1 | CXCL12, ID1 | [4, 5] |
| 2 | MMP2, WISP2 | [6, 7] |
| 3 | S100A4, BST2 | [8, 9] |
| 4 | LUM, FSTL1 | [10, 11] |
| 5 | PIEZO2 | [12] |
| 6 | RNASE1 | [13] |
| 7 | SCUBE3 | [14] |
| 8 | CENPM | [15] |

This table is generated from the public literature cited.

**Supplementary References**

1. Micutkova, L., et al., Insulin-like growth factor binding protein-6 delays replicative senescence of human fibroblasts. Mech Ageing Dev, 2011. 132(10): p. 468-79.

2. Valenzi, E., et al., Single-cell analysis reveals fibroblast heterogeneity and myofibroblasts in systemic sclerosis-associated interstitial lung disease. Ann Rheum Dis, 2019. 78(10): p. 1379-1387.

3. Liu, R., et al., SEMA3C Promotes Cervical Cancer Growth and Is Associated With Poor Prognosis. Front Oncol, 2019. 9: p. 1035.

4. Costa, A., et al., Fibroblast Heterogeneity and Immunosuppressive Environment in Human Breast Cancer. Cancer Cell, 2018. 33(3): p. 463-479.e10.

5. Edhayan, G., et al., Inflammatory properties of inhibitor of DNA binding 1 secreted by synovial fibroblasts in rheumatoid arthritis. Arthritis Res Ther, 2016. 18: p. 87.

6. Pattarayan, D., et al., Tannic acid modulates fibroblast proliferation and differentiation in response to pro-fibrotic stimuli. J Cell Biochem, 2018. 119(8): p. 6732-6742.

7. Micheletti, R., et al., The long noncoding RNA Wisper controls cardiac fibrosis and remodeling. Sci Transl Med, 2017. 9(395).

8. Li, Z., et al., Extracellular S100A4 as a key player in fibrotic diseases. J Cell Mol Med, 2020. 24(11): p. 5973-5983.

9. Yamamoto, A., et al., Transcription factor old astrocyte specifically induced substance is a novel regulator of kidney fibrosis. Faseb j, 2021. 35(2): p. e21158.

10. Barreto, G., et al., Lumican is upregulated in osteoarthritis and contributes to TLR4-induced pro-inflammatory activation of cartilage degradation and macrophage polarization. Osteoarthritis Cartilage, 2020. 28(1): p. 92-101.

11. Ni, S., et al., Follistatin-like protein 1 induction of matrix metalloproteinase 1, 3 and 13 gene expression in rheumatoid arthritis synoviocytes requires MAPK, JAK/STAT3 and NF-κB pathways. J Cell Physiol, 2018. 234(1): p. 454-463.

12. Lee, W., et al., Inflammatory signaling sensitizes Piezo1 mechanotransduction in articular chondrocytes as a pathogenic feed-forward mechanism in osteoarthritis. Proc Natl Acad Sci U S A, 2021. 118(13).

13. Zimmermann-Geller, B., et al., Influence of Extracellular RNAs, Released by Rheumatoid Arthritis Synovial Fibroblasts, on Their Adhesive and Invasive Properties. J Immunol, 2016. 197(7): p. 2589-97.

14. Yang, M., et al., Scube regulates synovial angiogenesis-related signaling. Med Hypotheses, 2013. 81(5): p. 948-53.

15. Mahmoud, A.D., et al., The Human-Specific and Smooth Muscle Cell-Enriched LncRNA SMILR Promotes Proliferation by Regulating Mitotic CENPF mRNA and Drives Cell-Cycle Progression Which Can Be Targeted to Limit Vascular Remodeling. Circ Res, 2019. 125(5): p. 535-551.

**Table S2.** Summary of bioactive molecular composition of raw materials.

| Raw Materials | Bioactive  Molecules | PubChem  CID | CAS | Molecular  Weight | Molecular  Formula |
| --- | --- | --- | --- | --- | --- |
| CB | Palmitic acid | 985 | 57-10-3 | 256.42 | C_16_H_32_O_2_ |
|  | Linoleic acid | 5280450 | 60-33-3 | 280.4 | C_18_H_32_O_2_ |
|  | Oleic acid | 445639 | 112-80-1 | 282.5 | C_18_H_34_O_2_ |
|  | Methyl pentadecanoate | 23518 | 7132-64-1 | 256.42 | C_16_H_32_O_2_ |
|  | Methyl hexadecatrienoate | 53438593 | 37822-81-4 | 264.4 | C_17_H_28_O_2_ |
|  | Methyl linoleate | 5284421 | 112-63-0 | 294.5 | C_19_H_34_O_2_ |
|  | Pterosin R | 46848401 | 76947-56-3 | 266.76 | C_15_H_19_ClO_2_ |
|  | Pterosin Y | 46848574 | 76947-59-6 | 280.32 | C_15_H_20_O_5_ |
|  | Aspidinol | 122841 | 519-40-4 | 224.25 | C_12_H_16_O_4_ |
|  | Vanillin | 1183 | 121-33-5 | 152.15 | C_8_H_8_O_3_ |
|  | Syringetin | 5281953 | 4423-37-4 | 346.3 | C_17_H_14_O_8_ |
|  | 4-Hydroxybenzoic acid | 135 | 99-96-7 | 138.12 | C_7_H_6_O_3_ |
|  | Acetovanillone | 2214 | 498-02-2 | 166.17 | C_9_H_10_O_3_ |
|  | Protocatehuic acid | 72 | 99-50-3 | 154.12 | C_7_H_6_O_4_ |
|  | Protocatechuic aldehyde | 8768 | 139-85-5 | 138.12 | C_7_H_6_O_3_ |
|  | CID 14562695 | 14562695 | 32885-82-8 | 278.34 | C_16_H_22_O_4_ |
|  | Caffeine | 2519 | 21399 | 194.19 | C_8_H_10_N_4_O_2_ |
|  | Kaempferol | 5280863 | 520-18-3 | 286.24 | C_15_H_10_O_6_ |
|  | n-Butyl-beta-d-fructopyranoside | 50914217 | 67884-27-9 | 236.26 | C_10_H_20_O_6_ |
| ED | Baohuoside I | 5488822 | 113558-15-9 | 514.5 | C_27_H_30_O_10_ |
|  | Baohuoside II | 5481982 | 55395-07-8 | 500.5 | C_26_H_28_O_10_ |
|  | Kaempferol-7-rhamnoside | 25079965 | 20196-89-8 | 432.4 | C_21_H_20_O_10_ |
|  | Icariin | 5318997 | 489-32-7 | 676.7 | C_33_H_40_O_15_ |
|  | Icariside I | 5745470 | 56725-99-6 | 530.5 | C_27_H_30_O_11_ |
|  | Sagittatoside A | 13916054 | 118525-35-2 | 676.7 | C_33_H_40_O_15_ |
|  | Sagittatoside B | 10146160 | 118525-36-3 | 646.6 | C_32_H_38_O_14_ |
|  | 2''-O-Rhamnosylicariside II | 5318987 | 135293-13-9 | 660.7 | C_33_H_40_O_14_ |
|  | Epimedoside A | 5317093 | 39012-04-9 | 662.6 | C_32_H_38_O_15_ |
|  | Maohuoside B | 131676049 | 849834-04-4 | 838.8 | C_39_H_50_O_20_ |
|  | Epimedin A | 254763566 | 110623-72-8 | 838.8 | C_39_H_50_O_20_ |
|  | Epimedin B | 5748393 | 110623-73-9 | 808.8 | C_38_H_48_O_19_ |
|  | Epimedin C | 5748394 | 110642-44-9 | 822.8 | C_39_H_50_O_19_ |
|  | Hydnocarpin | 5489114 | 51419-48-8 | 464.4 | C_25_H_20_O_9_ |
|  | 6-Demethoxycapillarisin | 5316511 | 61854-36-2 | 286.24 | C_15_H_10_O_6_ |
|  | Chlorogenic acid | 1794427 | 202650-88-2 | 354.31 | C_16_H_18_O_9_ |
|  | beta-Anhydroicaritin | 14583584 | 38226-86-7 | 368.4 | C_21_H_20_O_6_ |
|  | Coumalic acid | 68141 | 500-05-0 | 140.09 | C_6_H_4_O_4_ |
|  | Acuminatin | 6441048 | 41744-39-2 | 340.4 | C_21_H_24_O_4_ |
|  | Daidzein | 5281708 | 486-66-8 | 254.24 | C_15_H_10_O_4_ |
|  | Hyperoside | 5281643 | 482-36-0 | 464.4 | C_21_H_20_O_12_ |
|  | Maltol | 8369 | 118-71-8 | 126.11 | C_6_H_6_O_3_ |
|  | Icariside F2 | 14079045 | 115009-57-9 | 402.4 | C_18_H_26_O_10_ |
| CR | Cimigenol | 16020000 | 3779-59-7 | 488.7 | C_30_H_48_O_5_ |
|  | Cimigenoside | 16088242 | 27994-11-2 | 620.8 | C_35_H_56_O_9_ |
|  | Friedelin | 91472 | 559-74-0 | 426.7 | C_30_H_50_O |
|  | (+)-Sesamin dicatechol | 355102763 | 340167-81-9 | 330.3 | C_18_H_18_O_6_ |
|  | Ferulic acid | 445858 | 537-98-4 | 194.18 | C_10_H_10_O_4_ |
|  | Syringic acid | 10742 | 530-57-4 | 198.17 | C_9_H_10_O_5_ |
|  | beta-Sitosterol | 222284 | 83-46-5 | 414.7 | C_29_H_50_O |
| ZD | Butyl isobutyl phthalate | 28813 | 17851-53-5 | 278.34 | C_16_H_22_O_4_ |
|  | Hydroferulic acid | 14340 | 1135-23-5 | 196.2 | C_10_H_12_O_4_ |
|  | beta-Sitosterol | 222284 | 83-46-5 | 414.7 | C_29_H_50_O |
|  | Thymine | 1135 | 65-71-4 | 126.11 | C_5_H_6_N_2_O_2_ |
|  | 4-Hydroxybenzaldehyde | 126 | 123-08-0 | 122.12 | C_7_H_6_O_2_ |
| ABB | Ardisiacrispin A | 10328746 | 23643-61-0 | 1061.2 | C_52_H_84_O_22_ |
|  | Ardisiacrispin B | 10441164 | 112766-96-8 | 1075.2 | C_53_H_86_O_22_ |
|  | Euparone | 104654 | 53947-86-7 | 218.2 | C_12_H_10_O_4_ |
| CS | Oleanolic acid | 10494 | 508-02-1 | 456.7 | C_30_H_48_O_3_ |
|  | Ursolic acid | 64945 | 77-52-1 | 456.7 | C_30_H_48_O_3_ |
|  | Chlorogenic acid | 1794427 | 202650-88-2 | 354.31 | C_16_H_18_O_9_ |
|  | Cinnamic acid | 444539 | 140-10-3 | 148.16 | C_9_H_8_O_2_ |
|  | Caffeic acid | 689043 | 331-39-5 | 180.16 | C_9_H_8_O_4_ |
|  | Rutin | 5280805 | 153-18-4 | 610.5 | C_27_H_30_O_16_ |
|  | Quercetin | 5280343 | 117-39-5 | 302.23 | C_15_H_10_O_7_ |
|  | Hyperoside | 5281643 | 482-36-0 | 464.4 | C_21_H_20_O_12_ |
|  | 5-Propyl-2-thiouracil | 3002043 | 2954-52-1 | 170.23 | C_7_H_10_N_2_OS |
|  | Shikimic acid | 8742 | 138-59-0 | 174.15 | C_7_H_10_O_5_ |
|  | Citric acid | 311 | 77-92-9 | 192.12 | C_6_H_8_O_7_ |
|  | Gallic acid | 370 | 149-91-7 | 170.12 | C_7_H_6_O_5_ |
|  | beta-Sitosterol | 222284 | 83-46-5 | 414.7 | C_29_H_50_O |
|  | Sitogluside | 5742590 | 474-58-8 | 576.8 | C_35_H_60_O_6_ |
|  | 5-Hydroxymethylfurfural | 237332 | 67-47-0 | 126.11 | C_6_H_6_O_3_ |
|  | Kojic acid | 3840 | 501-30-4 | 142.11 | C_6_H_6_O_4_ |
|  | Cinchonine | 90454 | 118-10-5 | 294.4 | C_19_H_22_N_2_O |
|  | Nonacosan-10-ol | 25240035 | 504-55-2 | 424.8 | C_29_H_60_O |
|  | 1,2,4-Benzenetriol | 10787 | 533-73-3 | 126.11 | C_6_H_6_O_3_ |
| PL | Puerarin | 5281807 | 3681-99-0 | 416.4 | C_21_H_20_O_9_ |
|  | Daidzein | 5281708 | 486-66-8 | 254.24 | C_15_H_10_O_4_ |
|  | Daidzin | 107971 | 552-66-9 | 416.4 | C_21_H_20_O_9_ |
|  | Genistein | 5280961 | 446-72-0 | 270.24 | C_15_H_10_O_5_ |
|  | Biochanin A | 5280373 | 491-80-5 | 284.26 | C_16_H_12_O_5_ |
|  | Genistin | 5281377 | 529-59-9 | 432.4 | C_21_H_20_O_10_ |
|  | Formononetin | 5280378 | 485-72-3 | 268.26 | C_16_H_12_O_4_ |
|  | Ononin | 442813 | 486-62-4 | 430.4 | C_22_H_22_O_9_ |
|  | Pueraria glycoside | 5748205 | 117060-54-5 | 432.4 | C_21_H_20_O_10_ |
|  | Puerarin 6''-O-xyloside | 100990912 | 114240-18-5 | 548.5 | C_26_H_28_O_13_ |
|  | 3'-Methoxypuerarin | 5319485 | 117047-07-1 | 446.4 | C_22_H_22_O_10_ |
|  | Mirificin | 21676217 | 103654-50-8 | 548.5 | C_26_H_28_O_13_ |
|  | Isoliquiritigenin | 638278 | 961-29-5 | 256.25 | C_15_H_12_O_4_ |
|  | 3'-Methoxydaidzein | 5319422 | 21913-98-4 | 284.26 | C_16_H_12_O_5_ |
|  | Puerarin 4'-O-glucoside | 44257212 | 117047-08-2 | 578.5 | C_27_H_30_O_14_ |
|  | 3'-Methoxydaidzin | 10527347 | 200127-80-6 | 446.4 | C_22_H_22_O_10_ |
|  | Scoparone | 8417 | 120-08-1 | 206.19 | C_11_H_10_O_4_ |
|  | Coumestrol | 5281707 | 479-13-0 | 268.22 | C_15_H_8_O_5_ |
|  | Puerarol | 44257531 | unknow | 404.5 | C_25_H_24_O_5_ |
|  | Kuzubutenolide A | 10671648 | unknow | 460.4 | C_23_H_24_O_10_ |
|  | Lysine | 5962 | 56-87-1 | 146.19 | C_6_H_14_N_2_O_2_ |
|  | Phenylalanine | 6140 | 63-91-2 | 165.19 | C_9_H_11_NO_2_ |
|  | l-Isoleucine | 6306 | 443-79-8 | 131.17 | C_6_H_13_NO_2_ |
|  | Leucine | 6106 | 61-90-5 | 131.17 | C_6_H_13_NO_2_ |
|  | Histidine | 6274 | 71-00-1 | 155.15 | C_6_H_9_N_3_O_2_ |
|  | Allantoin | 204 | 97-59-6 | 158.12 | C_4_H_6_N_4_O_3_ |
|  | 5-Methylhydantoin | 69216 | 616-03-5 | 114.1 | C_4_H_6_N_2_O_2_ |
|  | Arachidic acid | 10467 | 506-30-9 | 312.5 | C_20_H_40_O_2_ |
|  | Anthraquinone | 6780 | 84-65-1 | 208.21 | C_14_H_8_O_2_ |
|  | Puemiricarpene | 10089353 | unknow | 352.4 | C_21_H_20_O_5_ |
| CL | Cyclocurcumin | 69879809 | 153127-42-5 | 368.4 | C_21_H_20_O_6_ |
|  | Bisdemethoxycurcumin | 5315472 | 33171-05-0 | 308.3 | C_19_H_16_O_4_ |
|  | Curcumin | 969516 | 458-37-7 | 368.4 | C_21_H_20_O_6_ |
|  | Demethoxycurcumin | 5469424 | 22608-11-3 | 338.4 | C_20_H_18_O_5_ |
|  | Methyl ferulate | 5357283 | 22329-76-6 | 208.21 | C_11_H_12_O_4_ |
|  | Vanillin | 1183 | 121-33-5 | 152.15 | C_8_H_8_O_3_ |
|  | Raspberry ketone | 21648 | 5471-51-2 | 164.2 | C_10_H_12_O_2_ |
|  | Zingerone | 31211 | 122-48-5 | 194.23 | C_11_H_14_O_3_ |
|  | Dehydrozingerone | 5354238 | 1080-12-2 | 192.21 | C_11_H_12_O_3_ |
|  | Zingerone | 31211 | 122-48-5 | 194.23 | C_11_H_14_O_3_ |
| PCL | Psoralen | 6199 | 66-97-7 | 186.16 | C_11_H_6_O_3_ |
|  | Methoxsalen | 4114 | 298-81-7 | 216.19 | C_12_H_8_O_4_ |
|  | 4'-Methoxyflavone | 77793 | 4143-74-2 | 252.26 | C_16_H_12_O_3_ |
|  | Apigenin | 5280443 | 520-36-5 | 270.24 | C_15_H_10_O_5_ |
|  | Bavachromene | 5321800 | 41743-38-8 | 322.4 | C_20_H_18_O_4_ |
|  | Isobavachromene | 5889042 | 56083-03-5 | 322.4 | C_20_H_18_O_4_ |
|  | Daidzein | 5281708 | 486-66-8 | 254.24 | C_15_H_10_O_4_ |
|  | Genistein | 5280961 | 446-72-0 | 270.24 | C_15_H_10_O_5_ |
|  | Biochanin A | 5280373 | 491-80-5 | 284.26 | C_16_H_12_O_5_ |
|  | 8-Prenyldaidzein | 14841119 | 135384-00-8 | 322.4 | C_20_H_18_O_4_ |
| CJB | Tangshenoside I | 6441191 | 117278-74-7 | 678.6 | C_29_H_42_O_18_ |
|  | Syringin | 5316860 | 118-34-3 | 372.4 | C_17_H_24_O_9_ |
|  | Apigenin dimethylether | 5281601 | 5128-44-9 | 298.29 | C_17_H_14_O_5_ |
|  | Taraxerol acetate | 94225 | 2189-80-2 | 468.8 | C_32_H_52_O_2_ |
|  | Friedelin | 91472 | 559-74-0 | 426.7 | C_30_H_50_O |

Certain bioactive molecules are present in multiple raw materials simultaneously.

**Supplementary References**

1. Jun, Y. and S. Nali, [Chemical Constituents from Clematidis Radix et Rhizoma]. Chinese Journal of Experimental Traditional Medical Formulae, 2017. 23(9): p. 41-45.

2. Lu, L., et al., [Advances in the Chemical Constituents and Pharmacological Studies of Epimedium]. Asia-Pacific Traditional Medicine, 2019. 15(16): p. 190-194.

3. Mengmeng, W., et al., [Chemical Constituents of Psoraleae Fructus and Its Main Toxic Ingredients]. Chinese Journal of Experimental Traditional Medical Formulae, 2019. 25(7): p. 207-209.

4. Pengying, L., et al., [Chemical Constituents of Guangdong Tu-Niu-Xi]. Acta Botanica Yunnanica, 2010. 32(2): p. 183-188.

5. Xin, L., et al., [Research Progress of Chemical Components and Pharmacological Action of Pueraria lobata]. Journal of Chinese Institute of Food Science and Technology, 2017. 17(9): p. 189-195.

6. Yan, Z. and Y. Haiyan, [Chemical constituents and pharmacological activities of the traditional Chinese medicine Chaenomelis Fructus: research advances]. J Int Pharm Res, 2019. 46(7): p. 507-515.

7. Yang, H., Q. Wu, and S. Yang, [Advances in research of chemical constituents and pharmacological activities of Cibotium barometz]. Chin J Exp Tradit Med Formulae, 2010. 15: p. 23-234.

8. Yuhan, C., et al., [Chemical constituents from rhizomes of Curcuma longa]. Chinese Traditional and Herbal Drugs, 2016. 47(7): p. 1074-1078.

**Supplementary Table 3.** Summary of potential targeting relationship pairs.

| Potential Targeting | Bioactive Molecules | Probability |
| --- | --- | --- |
| NPC1L1 | beta-Sitosterol | 0.8943 |
| NR1H3 | beta-Sitosterol | 0.7060 |
| PPARA | Methyl pentadecanoate | 0.6977 |
| FABP4 | Methyl pentadecanoate | 0.6707 |
| FABP3 | Methyl pentadecanoate | 0.6707 |
| FABP5 | Methyl pentadecanoate | 0.6707 |
| PPARD | Methyl pentadecanoate | 0.6707 |
| FABP2 | Methyl pentadecanoate | 0.6707 |
| FFAR1 | Methyl pentadecanoate | 0.6616 |
| NMUR2 | Rutin | 0.5646 |
| ADRA2A | Rutin | 0.5646 |
| ADRA2C | Rutin | 0.5646 |
| ACHE | Rutin | 0.5646 |
| RORC | beta-Sitosterol | 0.5644 |
| HMGCR | beta-Sitosterol | 0.5144 |
| PDE5A | Baohuoside I | 0.4725 |
| NOX4 | Hyperoside | 0.4693 |
| AKR1B1 | Hyperoside | 0.4693 |
| ADRA2C | Hyperoside | 0.4693 |
| CA2 | Hyperoside | 0.4693 |
| CA7 | Hyperoside | 0.4693 |
| CA12 | Hyperoside | 0.4693 |
| CA4 | Hyperoside | 0.4693 |
| RORC | Oleanolic acid | 0.4693 |
| POLB | Oleanolic acid | 0.4693 |
| PDE4D | Oleanolic acid | 0.4693 |
| PTPN1 | Oleanolic acid | 0.4693 |
| PTPRF | Oleanolic acid | 0.4693 |
| PTPN2 | Oleanolic acid | 0.4693 |
| HSD11B1 | Oleanolic acid | 0.4693 |
| PLA2G1B | Oleanolic acid | 0.4693 |
| CDC25B | Oleanolic acid | 0.4693 |
| ACP1 | Oleanolic acid | 0.4693 |
| AKR1B10 | Oleanolic acid | 0.4693 |
| RORC | Ursolic acid | 0.4693 |
| POLB | Ursolic acid | 0.4693 |
| PDE4D | Ursolic acid | 0.4693 |
| PTPN1 | Ursolic acid | 0.4693 |
| PTPRF | Ursolic acid | 0.4693 |
| PTPN2 | Ursolic acid | 0.4693 |
| HSD11B1 | Ursolic acid | 0.4693 |
| PLA2G1B | Ursolic acid | 0.4693 |
| CDC25B | Ursolic acid | 0.4693 |
| ACP1 | Ursolic acid | 0.4693 |
| AKR1B10 | Ursolic acid | 0.4693 |
| RORC | beta-Sitosterol | 0.4560 |
| SREBF2 | beta-Sitosterol | 0.4560 |
| AR | beta-Sitosterol | 0.4560 |
| NPC1L1 | beta-Sitosterol | 0.4560 |
| ESR1 | beta-Sitosterol | 0.4560 |
| ESR2 | beta-Sitosterol | 0.4560 |
| NR1H3 | beta-Sitosterol | 0.4560 |
| SHBG | beta-Sitosterol | 0.4560 |
| CYP17A1 | beta-Sitosterol | 0.4560 |
| HMGCR | beta-Sitosterol | 0.4560 |
| CYP51A1 | beta-Sitosterol | 0.4560 |
| CA7 | Puerarin | 0.4560 |
| CA12 | Puerarin | 0.4560 |
| ALDH2 | Daidzin | 0.4560 |
| IL2 | Daidzin | 0.4560 |
| PDE5A | Epimedoside A | 0.4401 |
| CA2 | Friedelin | 0.4326 |
| CA1 | Friedelin | 0.4326 |
| CA4 | Friedelin | 0.4326 |
| IL2 | Ononin | 0.4326 |
| NOX4 | Rutin | 0.4208 |
| AKR1B1 | Rutin | 0.4208 |
| CA2 | Rutin | 0.4208 |
| CA7 | Rutin | 0.4208 |
| CA12 | Rutin | 0.4208 |
| CA4 | Rutin | 0.4208 |
| PDE5A | Icariin | 0.4103 |
| ALDH2 | Ononin | 0.3924 |
| AKR1B1 | Chlorogenic acid | 0.3902 |
| AKR1B10 | Chlorogenic acid | 0.3902 |
| MAOA | Demethoxycurcumin | 0.3902 |
| APP | Demethoxycurcumin | 0.3902 |
| EP300 | Demethoxycurcumin | 0.3902 |
| PTGES | Demethoxycurcumin | 0.3902 |
| TLR9 | Demethoxycurcumin | 0.3902 |
| PDE5A | Baohuoside II | 0.3901 |
| IL2 | Sitogluside | 0.3864 |
| CYP51A1 | beta-Sitosterol | 0.3810 |
| ALDH2 | Daidzein | 0.3792 |
| ESR1 | Daidzein | 0.3792 |
| CA7 | Daidzein | 0.3792 |
| ESR2 | Daidzein | 0.3792 |
| CA12 | Daidzein | 0.3792 |
| CA4 | Daidzein | 0.3792 |
| AKR1B1 | Isoliquiritigenin | 0.3792 |
| CHRNA7 | Isoliquiritigenin | 0.3792 |
| ABCC1 | 4'-Methoxyflavone | 0.3792 |
| ABCG2 | 4'-Methoxyflavone | 0.3792 |
| TNKS | 4'-Methoxyflavone | 0.3792 |
| ALDH2 | 8-Prenyldaidzein | 0.3792 |
| ESR1 | 8-Prenyldaidzein | 0.3792 |
| CA7 | 8-Prenyldaidzein | 0.3792 |
| ESR2 | 8-Prenyldaidzein | 0.3792 |
| CA12 | 8-Prenyldaidzein | 0.3792 |
| CA4 | 8-Prenyldaidzein | 0.3792 |
| MAOA | Curcumin | 0.3773 |
| APP | Curcumin | 0.3773 |
| EP300 | Curcumin | 0.3773 |
| PTGES | Curcumin | 0.3773 |
| TLR9 | Curcumin | 0.3773 |
| FABP4 | Palmitic acid | 0.3710 |
| PPARA | Palmitic acid | 0.3710 |
| FABP3 | Palmitic acid | 0.3710 |
| FABP5 | Palmitic acid | 0.3710 |
| PPARD | Palmitic acid | 0.3710 |
| FFAR1 | Palmitic acid | 0.3710 |
| FABP2 | Palmitic acid | 0.3710 |
| ACHE | Hyperoside | 0.3688 |
| NQO2 | Hyperoside | 0.3688 |
| TNF | 3'-Methoxydaidzin | 0.3638 |
| IL2 | 3'-Methoxydaidzin | 0.3638 |
| CYP17A1 | beta-Sitosterol | 0.3561 |
| NMUR2 | Hyperoside | 0.3520 |
| ADRA2A | Hyperoside | 0.3520 |
| FABP4 | Linoleic acid | 0.3463 |
| PTGS1 | Linoleic acid | 0.3463 |
| PPARG | Linoleic acid | 0.3463 |
| PPARA | Linoleic acid | 0.3463 |
| FABP3 | Linoleic acid | 0.3463 |
| PPARD | Linoleic acid | 0.3463 |
| FFAR1 | Linoleic acid | 0.3463 |
| FABP4 | Oleic acid | 0.3463 |
| FAAH | Oleic acid | 0.3463 |
| PPARG | Oleic acid | 0.3463 |
| PPARA | Oleic acid | 0.3463 |
| TERT | Oleic acid | 0.3463 |
| FABP3 | Oleic acid | 0.3463 |
| FABP5 | Oleic acid | 0.3463 |
| PPARD | Oleic acid | 0.3463 |
| FABP1 | Oleic acid | 0.3463 |
| ESR1 | Coumestrol | 0.3463 |
| ESR2 | Coumestrol | 0.3463 |
| TBXAS1 | Genistein | 0.3463 |
| MAOA | Genistein | 0.3463 |
| EGFR | Genistein | 0.3463 |
| ESR1 | Genistein | 0.3463 |
| MGAM | Genistein | 0.3463 |
| HTR2A | Genistein | 0.3463 |
| HTR2C | Genistein | 0.3463 |
| ADORA1 | Genistein | 0.3463 |
| ESR2 | Genistein | 0.3463 |
| ADORA2A | Genistein | 0.3463 |
| HSD17B1 | Genistein | 0.3463 |
| ESRRA | Genistein | 0.3463 |
| ESRRB | Genistein | 0.3463 |
| ABCG2 | Genistein | 0.3463 |
| IL2 | Formononetin | 0.3463 |
| NOX4 | Apigenin | 0.3463 |
| AKR1B1 | Apigenin | 0.3463 |
| CDK5R1 | Apigenin | 0.3463 |
| XDH | Apigenin | 0.3463 |
| MAOA | Apigenin | 0.3463 |
| FLT3 | Apigenin | 0.3463 |
| CYP19A1 | Apigenin | 0.3463 |
| ESR1 | Apigenin | 0.3463 |
| CCNB3 | Apigenin | 0.3463 |
| ACHE | Apigenin | 0.3463 |
| ADORA1 | Apigenin | 0.3463 |
| PTGS2 | Apigenin | 0.3463 |
| ESR2 | Apigenin | 0.3463 |
| CDK6 | Apigenin | 0.3463 |
| ADORA2A | Apigenin | 0.3463 |
| SYK | Apigenin | 0.3463 |
| GSK3B | Apigenin | 0.3463 |
| ABCC1 | Apigenin | 0.3463 |
| HSD17B1 | Apigenin | 0.3463 |
| TTR | Apigenin | 0.3463 |
| CSNK2A1 | Apigenin | 0.3463 |
| CFTR | Apigenin | 0.3463 |
| CYP1B1 | Apigenin | 0.3463 |
| ABCG2 | Apigenin | 0.3463 |
| AKR1B10 | Apigenin | 0.3463 |
| TNKS2 | Apigenin | 0.3463 |
| TNKS | Apigenin | 0.3463 |
| TBXAS1 | Daidzein | 0.3463 |
| MAOA | Daidzein | 0.3463 |
| EGFR | Daidzein | 0.3463 |
| MGAM | Daidzein | 0.3463 |
| HTR2A | Daidzein | 0.3463 |
| HTR2C | Daidzein | 0.3463 |
| ADORA1 | Daidzein | 0.3463 |
| ADORA2A | Daidzein | 0.3463 |
| HSD17B1 | Daidzein | 0.3463 |
| ESRRA | Daidzein | 0.3463 |
| ESRRB | Daidzein | 0.3463 |
| ABCG2 | Daidzein | 0.3463 |
| PSEN2 | cimigoside | 0.3407 |
| NOX4 | Quercetin | 0.3405 |
| MAPT | Quercetin | 0.3405 |
| KDM4E | Quercetin | 0.3405 |
| GPR35 | Quercetin | 0.3405 |
| AVPR2 | Quercetin | 0.3405 |
| TOP2A | Quercetin | 0.3405 |
| AKR1B1 | Quercetin | 0.3405 |
| XDH | Quercetin | 0.3405 |
| MAOA | Quercetin | 0.3405 |
| IGF1R | Quercetin | 0.3405 |
| FLT3 | Quercetin | 0.3405 |
| CYP19A1 | Quercetin | 0.3405 |
| INSR | Quercetin | 0.3405 |
| EGFR | Quercetin | 0.3405 |
| F2 | Quercetin | 0.3405 |
| CA2 | Quercetin | 0.3405 |
| PIM1 | Quercetin | 0.3405 |
| ALOX5 | Quercetin | 0.3405 |
| AURKB | Quercetin | 0.3405 |
| DRD4 | Quercetin | 0.3405 |
| ACHE | Quercetin | 0.3405 |
| ADORA1 | Quercetin | 0.3405 |
| CA7 | Quercetin | 0.3405 |
| GLO1 | Quercetin | 0.3405 |
| MYLK | Quercetin | 0.3405 |
| MPO | Quercetin | 0.3405 |
| PIK3R1 | Quercetin | 0.3405 |
| ADORA2A | Quercetin | 0.3405 |
| DAPK1 | Quercetin | 0.3405 |
| PYGL | Quercetin | 0.3405 |
| SYK | Quercetin | 0.3405 |
| CA1 | Quercetin | 0.3405 |
| GSK3B | Quercetin | 0.3405 |
| SRC | Quercetin | 0.3405 |
| PTK2 | Quercetin | 0.3405 |
| HSD17B2 | Quercetin | 0.3405 |
| KDR | Quercetin | 0.3405 |
| MMP13 | Quercetin | 0.3405 |
| MMP3 | Quercetin | 0.3405 |
| CA3 | Quercetin | 0.3405 |
| ALOX15 | Quercetin | 0.3405 |
| ABCC1 | Quercetin | 0.3405 |
| PLK1 | Quercetin | 0.3405 |
| CA6 | Quercetin | 0.3405 |
| CDK1 | Quercetin | 0.3405 |
| MMP9 | Quercetin | 0.3405 |
| CA12 | Quercetin | 0.3405 |
| PIK3CG | Quercetin | 0.3405 |
| MMP2 | Quercetin | 0.3405 |
| PKN1 | Quercetin | 0.3405 |
| CA14 | Quercetin | 0.3405 |
| CA9 | Quercetin | 0.3405 |
| CSNK2A1 | Quercetin | 0.3405 |
| ALOX12 | Quercetin | 0.3405 |
| MET | Quercetin | 0.3405 |
| CA4 | Quercetin | 0.3405 |
| NEK2 | Quercetin | 0.3405 |
| CXCR1 | Quercetin | 0.3405 |
| CAMK2B | Quercetin | 0.3405 |
| ALK | Quercetin | 0.3405 |
| AKT1 | Quercetin | 0.3405 |
| ABCB1 | Quercetin | 0.3405 |
| NEK6 | Quercetin | 0.3405 |
| PLA2G1B | Quercetin | 0.3405 |
| CA5A | Quercetin | 0.3405 |
| BACE1 | Quercetin | 0.3405 |
| CYP1B1 | Quercetin | 0.3405 |
| AXL | Quercetin | 0.3405 |
| ABCG2 | Quercetin | 0.3405 |
| APEX1 | Quercetin | 0.3405 |
| NUAK1 | Quercetin | 0.3405 |
| AKR1C2 | Quercetin | 0.3405 |
| AKR1C1 | Quercetin | 0.3405 |
| AKR1C3 | Quercetin | 0.3405 |
| AKR1C4 | Quercetin | 0.3405 |
| CA13 | Quercetin | 0.3405 |
| AKR1A1 | Quercetin | 0.3405 |
| FABP4 | Arachidic acid | 0.3405 |
| PPARA | Arachidic acid | 0.3405 |
| FABP3 | Arachidic acid | 0.3405 |
| FABP5 | Arachidic acid | 0.3405 |
| PPARD | Arachidic acid | 0.3405 |
| FFAR1 | Arachidic acid | 0.3405 |
| FABP2 | Arachidic acid | 0.3405 |
| MMP13 | Chlorogenic acid | 0.3398 |
| MMP2 | Chlorogenic acid | 0.3398 |
| MMP12 | Chlorogenic acid | 0.3398 |
| VEGFA | Icariside F2 | 0.3371 |
| FGF1 | Icariside F2 | 0.3371 |
| FGF2 | Icariside F2 | 0.3371 |
| HPSE | Icariside F2 | 0.3371 |
| BACE1 | Demethoxycurcumin | 0.3315 |
| ALOX5 | (+)-Sesamin dicatechol | 0.3307 |
| ODC1 | Bavachromene | 0.3307 |
| ODC1 | Isobavachromene | 0.3307 |
| CDC25B | Anthraquinone | 0.3292 |
| ACHE | Psoralen | 0.3292 |
| CYP1A2 | Psoralen | 0.3292 |
| RPS6KA3 | Hyperoside | 0.3269 |
| RPS6KA3 | Rutin | 0.3249 |
| XDH | Syringetin | 0.3232 |
| CA2 | Syringetin | 0.3232 |
| CA7 | Syringetin | 0.3232 |
| CA12 | Syringetin | 0.3232 |
| CA4 | Syringetin | 0.3232 |
| CYP1B1 | Syringetin | 0.3232 |
| BACE1 | Curcumin | 0.3213 |
| CYP19A1 | Biochanin A | 0.3202 |
| CA7 | Biochanin A | 0.3202 |
| HSD17B2 | Biochanin A | 0.3202 |
| CA12 | Biochanin A | 0.3202 |
| CA4 | Biochanin A | 0.3202 |
| CBR1 | Biochanin A | 0.3202 |
| NQO2 | Rutin | 0.3153 |
| CYP19A1 | beta-Sitosterol | 0.3144 |
| SREBF2 | beta-Sitosterol | 0.3144 |
| CA2 | Gallic acid | 0.3085 |
| CA7 | Gallic acid | 0.3085 |
| CA1 | Gallic acid | 0.3085 |
| CA3 | Gallic acid | 0.3085 |
| CA6 | Gallic acid | 0.3085 |
| CA12 | Gallic acid | 0.3085 |
| CA14 | Gallic acid | 0.3085 |
| CA9 | Gallic acid | 0.3085 |
| FUT7 | Gallic acid | 0.3085 |
| CA4 | Gallic acid | 0.3085 |
| CA5B | Gallic acid | 0.3085 |
| CA5A | Gallic acid | 0.3085 |
| CA13 | Gallic acid | 0.3085 |
| CACNA2D1 | Phenylalanine | 0.3085 |
| PDE5A | 2''-O-Rhamnosylicariside II | 0.3061 |
| TBXAS1 | Daidzein | 0.3006 |
| MAOA | Daidzein | 0.3006 |
| EGFR | Daidzein | 0.3006 |
| MGAM | Daidzein | 0.3006 |
| HTR2A | Daidzein | 0.3006 |
| HTR2C | Daidzein | 0.3006 |
| ADORA1 | Daidzein | 0.3006 |
| ADORA2A | Daidzein | 0.3006 |
| HSD17B1 | Daidzein | 0.3006 |
| ESRRA | Daidzein | 0.3006 |
| ESRRB | Daidzein | 0.3006 |
| ABCG2 | Daidzein | 0.3006 |
| TBXAS1 | Genistein | 0.3006 |
| MAOA | Genistein | 0.3006 |
| EGFR | Genistein | 0.3006 |
| MGAM | Genistein | 0.3006 |
| HTR2A | Genistein | 0.3006 |
| HTR2C | Genistein | 0.3006 |
| ADORA1 | Genistein | 0.3006 |
| ADORA2A | Genistein | 0.3006 |
| HSD17B1 | Genistein | 0.3006 |
| ESRRA | Genistein | 0.3006 |
| ESRRB | Genistein | 0.3006 |
| ABCG2 | Genistein | 0.3006 |
| TBXAS1 | 8-Prenyldaidzein | 0.3006 |
| MAOA | 8-Prenyldaidzein | 0.3006 |
| EGFR | 8-Prenyldaidzein | 0.3006 |
| MGAM | 8-Prenyldaidzein | 0.3006 |
| HTR2A | 8-Prenyldaidzein | 0.3006 |
| HTR2C | 9-Prenyldaidzein | 0.3006 |
| ADORA1 | 10-Prenyldaidzein | 0.3006 |
| ADORA2A | 11-Prenyldaidzein | 0.3006 |
| HSD17B1 | 12-Prenyldaidzein | 0.3006 |
| ESRRA | 13-Prenyldaidzein | 0.3006 |
| ESRRB | 14-Prenyldaidzein | 0.3006 |
| ABCG2 | 15-Prenyldaidzein | 0.3006 |
